# Effects of Density and Food Availability on the Growth of Captive‐Reared Eastern Hellbenders

**DOI:** 10.1002/zoo.70087

**Published:** 2026-06-15

**Authors:** Shelby M. Royal, Summer S. Brown, Nicholas G. Burgmeier, Rod N. Williams, Jason T. Hoverman

**Affiliations:** ^1^ Department of Forestry and Natural Resources Purdue University West Lafayette Indiana USA; ^2^ Office of the Provost Texas Tech University Lubbock Texas USA

**Keywords:** amphibian, amphibian declines, body condition, captive management, competition, reintroduction, salamander

## Abstract

Nascent captive rearing programs often lack empirical data to inform the development of husbandry practices. This lack of data, especially for density and food availability recommendations, can lead to suboptimal husbandry standards that can impair growth or survival. We conducted two experiments with captive‐reared Eastern Hellbenders (*Cryptobranchus alleganiensis alleganiensis*) to explore the effects of husbandry conditions on growth. In the first experiment, we examined growth after 7 months when reared at three densities (low, medium, or high). We found no differences in growth among the treatments. In the second experiment, we examined the interactive effects of density and food availability on growth over 8 months. The experiment was a 3 × 3 factorial design with three densities (low, medium, and high) and three food availability treatments (low, medium, and high). We found a significant effect of food availability on growth during the experiment, but no effect of density or interactive effects. Averaged across the density treatments, we found that growth was 26%–46% greater in the high food availability treatment compared to the low and medium food availability treatments. This work suggests that food is the major factor limiting captive‐reared Hellbender growth and that increasing rearing density to the tested levels does not negatively impact growth. Implementing these changes may result in larger, more robust Hellbenders and increase the success of reintroduction programs.

## Summary

- For captive‐reared Hellbenders, food rather than density is the major factor limiting growth
- Increasing rearing density to the tested levels may result in larger, more robust Hellbenders, and increase the success of reintroduction programs

## Graphical abstract

Our experiments demonstrate that food is the major factor limiting captive‐reared Hellbender growth and that increasing rearing density to the tested levels does not negatively impact growth. Implementing these changes may result in larger, more robust Hellbenders and increase the success of reintroduction programs.

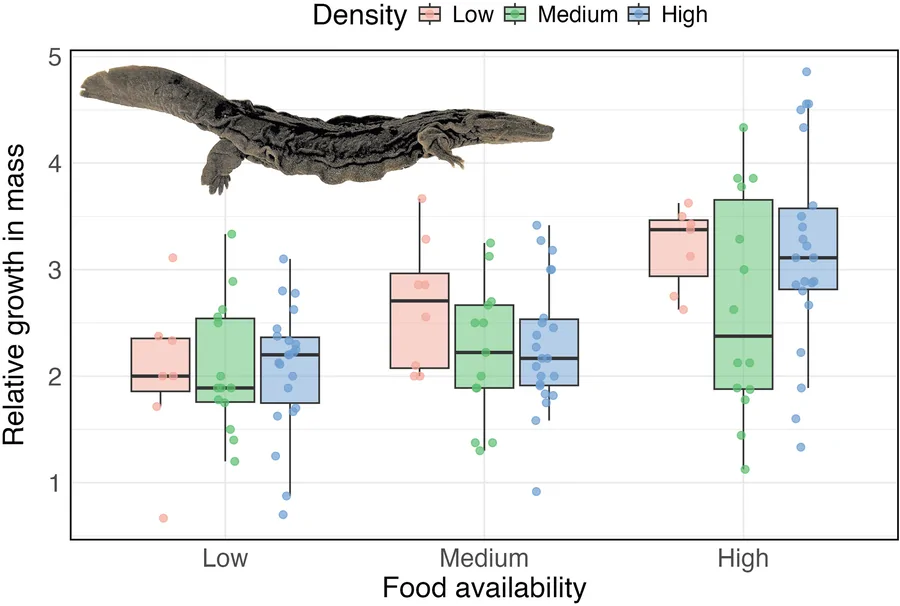

## 1 Introduction

Amphibians are the most imperiled vertebrate group, with ~41% of known species considered threatened or endangered (Luedtke et al. 2023; Stuart et al. 2004). In response to their conservation status, captive‐rearing programs have been implemented to reduce extirpation or extinction risk (Scott et al. 2005). One advantage of amphibian captive‐rearing programs is the controlled system to study young age classes and document responses to rearing conditions, which can be challenging with *in situ* approaches where mortality rates can be high (Goldberg et al. 2012).

In response to a broad range of environmental factors (e.g., predation, competition), amphibians express phenotypic variation in morphology, behavior, growth, and development (Newman 1994a; Relyea 2001, 2002; Van Buskirk 2001). Competition, in particular, has been well studied in amphibian systems. For instance, many tadpoles express greater activity, larger bodies, and longer intestines when reared at higher densities (Relyea 2002; Relyea and Auld 2004, 2005). These traits increase foraging rates and food processing efficiency in the presence of competitors. In some salamander and frog species, larvae can develop into a cannibalistic morph at high population densities when food is limited, or body size distributions are skewed (Browne et al. 2003; Morey and Reznick 2001; Newman 1994b; Slembrouck et al. 2009). While there is a rich literature examining competition in amphibians, its application to captive management programs remains limited (Collins and Cheek 1983; Tornabene and Hoverman 2018; Wildy et al. 2001). A major goal of captive‐rearing programs is to provide enough food and space to produce large, healthy individuals while minimizing body size variation. Husbandry recommendations (i.e. density and food availability) for some species are not well understood and often follow folklore husbandry approaches (Arbuckle 2013; Harvey Pough. 2007). The lack of empirical data addressing the effects of density and food availability on growth likely result in suboptimal husbandry conditions (Harvey Pough. 2007).

Eastern Hellbenders (*Cryptobranchus alleganiensis alleganiensis*; hereafter, Hellbenders) are fully aquatic salamanders that are in severe decline throughout most of their native range in the eastern United States due to habitat degradation and poor water quality of rivers and streams (Bodinof et al. 2012; Burgmeier et al. 2011; Williams et al. 1981). Because of these declines, captive‐rearing and reintroduction programs have been developed throughout the United States to improve the survival of younger age classes (Kenison and Williams 2018; Petranka 1989). Unfortunately, little information is known about the pre‐adult life history and biology of the Hellbenders. Managing Hellbenders requires an adaptive approach and a deeper understanding of the range of rearing conditions that affect their overall survival (Kenison and Williams 2018; Williams et al. 1981).

While Hellbenders are reared in the thousands in captive‐rearing and reintroduction programs across 15 states (Burgmeier et al. 2017; Kaunert et al. 2024; Macklem et al. 2025), little formal research is conducted in these facilities to advance our knowledge of general husbandry needs for this imperiled species. Captive‐rearing programs for Hellbenders are not standardized, which could potentially lead to inefficiencies and impact conservation efforts. Guidelines for captive‐rearing densities are currently determined by the size of the enclosure and the mass of the Hellbender; however, these guidelines are only proxy numbers. In captive settings, there is likely a specific density and food availability requirement necessary to minimize cannibalism and aggression, while optimizing growth rates. However, it is unclear if Hellbender densities or the amount of available food result in functional responses that can impact growth trajectories.

We conducted two experiments with captive‐reared Hellbenders to explore the effects of husbandry conditions on growth. In the first experiment, we examined Hellbender growth and body condition after 7 months when reared at three densities (low, medium, or high) and fed 1 g of food per individual per feeding. We hypothesized that increasing density would not affect Hellbender growth or body condition because per capita food resources would be maintained. We conducted a second follow‐up study to examine the interactive effects of density and food availability on growth over 8 months. Our experiment was a 3 × 3 factorial design with three densities (low, medium, and high) and three food availability treatments (low, medium, and high). We hypothesized that higher food availability would increase Hellbender growth and body condition, but density treatments would have no effect.

## 2 Materials and Methods

### 2.1 Experiment 1: Density Effects

The first experiment was conducted at Purdue University's Aquaculture Research Lab (ARL). In 2018, we collected 330 Eastern Hellbender eggs from a small stream in eastern Kentucky (location omitted due to sensitivity of the site). From the 296 hellbenders that survived to age 3, we randomly selected 102 hellbenders for the experiment. These individuals were size‐sorted by mass to reduce intraspecific aggression. Prior to beginning the experiment, we used spot pattern mapping for unique identification of individuals (Kenison and Williams 2018). Hellbenders were placed in a clear Lexan box with white sheets of paper placed underneath for a consistent background. We used a Nikon D7500 with a Nikon DX 18–105 mm lens (Nikon Corporation, Tokyo, Japan), at a height of 20.32 cm from the lens (Sannolo et al. 2016) and attached to a ring light (color temperature of 5000 K and a brightness of 10) to provide consistent and ample lighting. We also measured each Hellbender's mass (g), snout‐vent length (cm), and total length (cm). We measured mass using a Medium Tote Tray (Nasco, W05175) placed on a Washdown Compact Scale (A&D, SK‐2000WPZ). Length was measured by placing individuals in a V‐shaped holding device that had a ruler glued to the side for measurements (Kenison and Williams 2018).

For the experiment, Hellbenders were housed in raceway tanks (177.8 cm × 40.64 cm × 20.57 cm) within a walk‐in environmental chamber. We set the lights in the environmental chamber on a 14:10 light: dark cycle and water temperature of 15°C. The floor area of each raceway tank was 7225.79 cm^2^. Inside each tank were two uniform concrete huts for shelter (Figure 1; Macke et al. 2022). The entrance height of the huts was 3.81 cm, and the cavity volume was 9315.9 cm^3^. The experimental design consisted of three density treatments, each replicated twice for six total raceway tanks. The raceways held 9 (802.87 cm^2^ per hellbender), 15 (481.72 cm^2^ per hellbender), or 22 individuals (328.45 cm^2^ per hellbender) for low, medium, and high densities, respectively. These densities were selected based on the current guideline of 15 Ozark hellbenders in a 78‐gallon tank (Ettling et al. 2013). The raceway tanks were stacked, with three rows of two tanks. All tanks were on the same flow‐through system. At the beginning of the experiment, the mean mass ± SD was 34.3 g ± 7.2 g, 23.9 g ± 5.8 g, and 30.9 g ± 6.8 g for the low, medium, and high densities, respectively.

**Figure 1:**
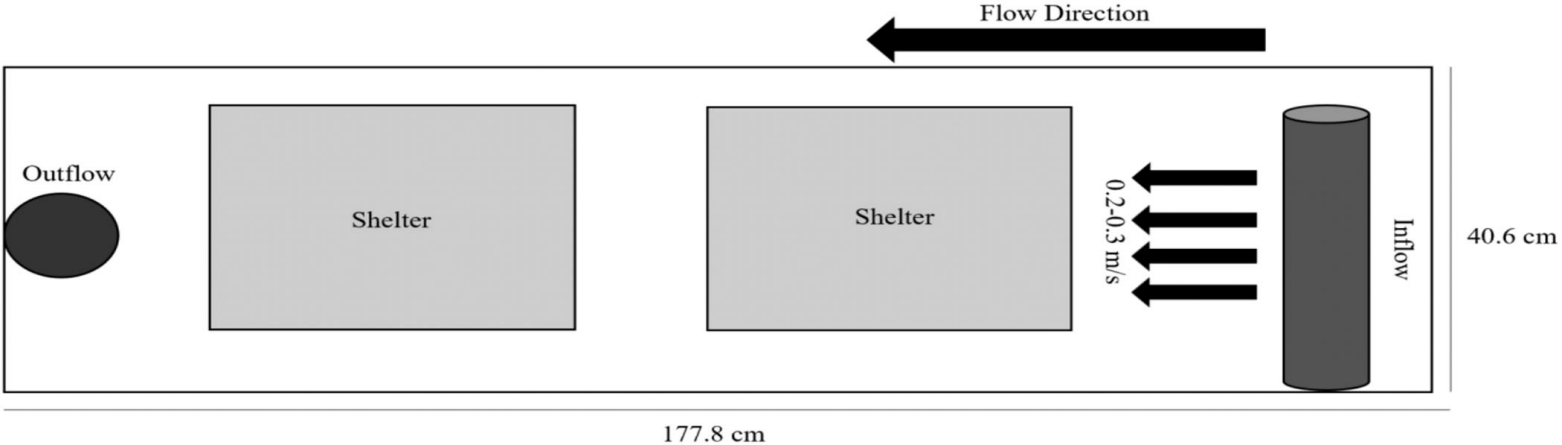
Schematic of Eastern Hellbender (*Cryptobranchus alleganiensis alleganiensis*) raceway tank used in Experiment 1 at Purdue Aquaculture Research Laboratory. Inside each tank were two uniform concrete huts for shelter. The flow rate within the tank ranged from 0.2 to 0.3 m/s. There were six replicate tanks used in the experiment.

We fed the Hellbenders frozen mysis shrimp (*Mysida* sp.) on Mondays and bloodworms (*Chironomidae* sp.) on Wednesdays and Fridays. We thawed and measured 1 g of shrimp or bloodworms per Hellbender for each tank. Their food was placed in two feeding rings (5.08 cm PVC pipe) in the center of the raceway tank. The feeding rings helped keep the food from dispersing throughout the tank.

Our experiment ran from November 2021 to July 2022. At the conclusion of the experiment, we collected mass (g), snout‐vent length (cm), and total length (cm) for each individual and uniquely identified each of them using their original spot patterns. One individual died during the experiment and was excluded from the analyses. We used three Pearson correlation tests to determine if mass, snout‐vent length, and total length were significantly correlated. We found that all three variables were significantly correlated (*r* ^2^ > 0.65, *p* < 0.001). Thus, we chose to focus our analysis on mass because it is the morphometric measurement most commonly used for captive management and Hellbender transmitter surgery studies (Bodinof et al. 2012). We calculated relative growth in mass as the difference between the final and initial mass divided by the initial mass. We then tested for the effects of density on relative growth using generalized linear mixed models with tank as a random effect. The analysis was conducted in R 4.4.2 (R Development Core Team 2024) using the lme4 package. We confirmed homogeneity of variance using Bartlett's Test (*K* ^2^ = 2.8, df = 2, *p* = 0.249).

We also calculated the scaled mass index (SMI) to assess body condition across the density treatments. SMI is assumed to be the best indicator for assessing amphibian body condition and was calculated using the following equation from Peig and Green 2009):

$${\hat{M}}_{i}=M_{i}{\left[\frac{L_{o}}{L_{i}}\right]}^{b_{SMA}}$$

where *M*_*i*_ and *L*_*i*_ are final body mass and final snout‐vent length of individual *i*, respectively; ^*b*^ *SMA* is a scaling exponent estimated from calculations in the regression of ln(mass) against ln(snout‐vent length); *L*_*0*_ is the calculated mean snout‐vent length across all individuals; and ${\hat{M}}_{i}$is the estimated body mass of individual *i* when snout‐vent length is standardized to *L*_*0*_. We then tested for the effects of density on SMI using generalized linear mixed models with tank as a random effect. The analysis was conducted in R 4.4.2 (R Development Core Team 2024) using the lme4 package. We confirmed homogeneity of variance using Bartlett's Test (*K* ^2^ = 2.1, df = 2, *p* = 0.357).

### 2.2 Experiment 2: Interactive Effects of Density and Food Availability

Hellbenders were collected as a clutch of 600 eggs from eastern Kentucky, USA in 2020 and were reared for 1 year at Mesker Park Zoo and Botanic Garden in Evansville, IN, USA before being transferred to ARL in August 2021 for the experiment. We placed Hellbenders in an environmental chamber within ARL to control environmental factors (i.e., lighting and water temperature). We set the lights in the environmental chamber to a 14:10 light: dark cycle and water temperature of 15°C. We reared Hellbenders in a partial recirculation and flow‐through system using 95‐liter polyethylene aquaria (68.85 cm × 45.71 cm × 30.48 cm) with PVC hides and no substrate or current. Our 18 aquaria were divided into three systems (6 aquaria per system) and shared a single water source. We used 132, one‐and‐a‐half‐year‐old Hellbenders with an average mass of 9 g ± 1.5 g (mean ± SD) and total body length of 10 cm ± 0.8 cm (mean ± SD). Individuals were identified using spot pattern mapping as described above (Kenison and Williams 2018).

Our experiment was a factorial combination of three density treatments (low, medium, or high) crossed with three food availability treatments (low, medium, or high). These nine treatments were replicated twice for a total of 18 aquaria (Figure 2). Individuals were initially sorted by mass to reduce intraspecific competition and aggression and minimize differences in starting mass. We added 4, 7, or 11 larvae per tank for low (786.78 cm^2^ per hellbender), medium (449.59 cm^2^ per hellbender), and high density (286.10 cm^2^ per hellbender) treatments, respectively. We determined the high and low densities based on the estimated maximum number of 2‐year‐old Ozark Hellbenders recommended in 95‐liter aquaria (below 60 g for a maximum of 11, above 60 g for a maximum of 4; Ettling et al. 2013). We determined the medium density by taking the average of the high and low densities.

**Figure 2:**
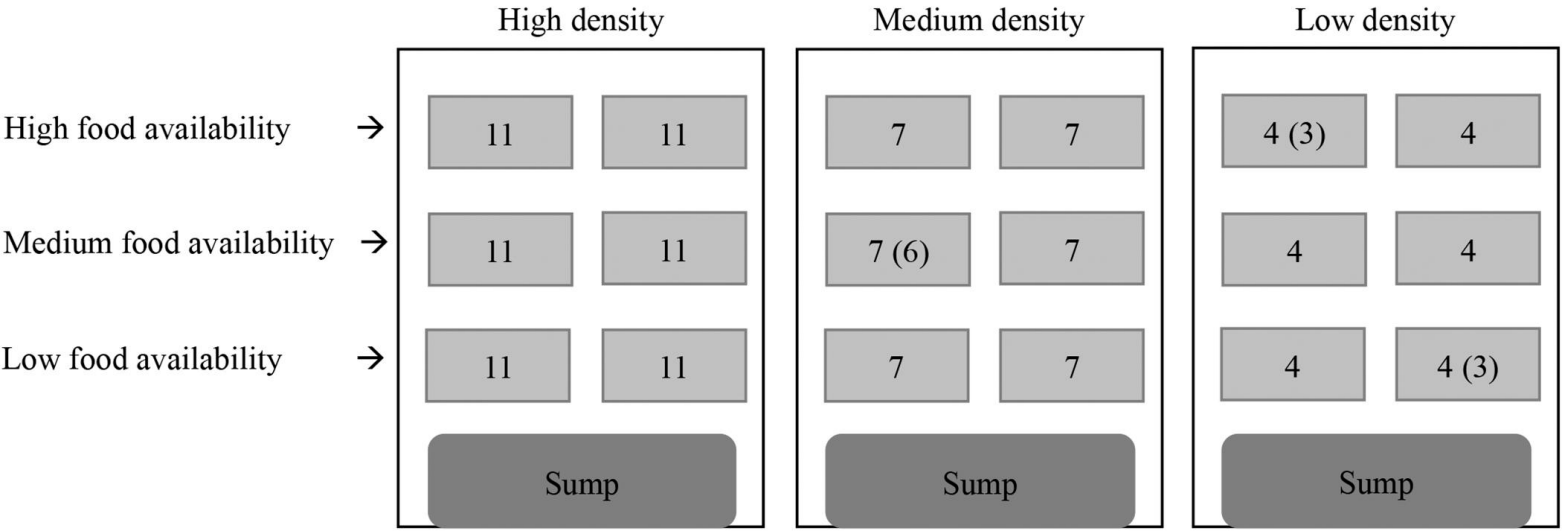
Schematic of the experimental design for Experiment 2 with Eastern Hellbenders (*Cryptobranchus alleganiensis alleganiensis*) at Purdue Aquaculture Research Laboratory. The three systems were divided by the density treatments, with the food availability treatments assigned within each system. Numbers represent the starting number of Hellbenders in the tank, and the number in parentheses indicates the final number in the tanks at the conclusion of the experiment. Schematic not to scale.

The food availability treatments were designed to provide a specific food ration based on the average body mass of the Hellbenders in the tanks. Thus, the low, medium, and high food availability treatments were assigned a ration of 5.6%, 8.3%, and 11.1% of body mass per individual per feeding, respectively. The low availability treatment was selected based on husbandry standards obtained from several zoos rearing Hellbenders. Using this estimate, the medium and high availability treatments were selected to be roughly 50% and 100% higher, respectively, compared to the low treatment. The Hellbenders were weighed every 2 months to adjust the ration and account for growth during the experiment. We fed Hellbenders a variety of food items three times per week: mysis (*Mysis* sp.) on Mondays and bloodworms (*Glycera* sp.) on Wednesdays and Fridays.

Over the 8‐month experiment, we had three mortalities (one each in the high food‐medium density, high food‐low density, and low food‐low density treatments), and these were excluded from analyses. Our response variables were relative growth in mass and SMI, which were calculated as described above. We used linear mixed effects models to examine the effects of density, food availability, and their interaction on our response variables using the lme4 package in R 4.4.2 (R Development Core Team 2024). Tank was included as a random effect. Pair‐wise comparisons were conducted using Tukey's Honestly Significant Difference (Tukey HSD) post‐hoc test. We confirmed homogeneity of variance using Bartlett's Test (relative growth in mass, *K* ^2^ = 14.8, df = 8, *p* = 0.064; SMI, *K* ^2^ = 5.0, df = 8, *p* = 0.760).

Artificial Intelligence‐generated content tools were not used for the manuscript.

## 3 Results

### 3.1 Experiment 1. Density Effects

The density treatments had no effect on relative growth in mass (*F*_2,88_ = 1.1; *p* = 0.353) or SMI (*F*_2,88_ = 1.2; *p* = 0.359) for the hellbenders (Figure 3). Averaged across the experiment, Hellbenders grew by 83% and had an SMI of 51. The full dataset is available in the Supporting Information S1 and S2: Experiments 1 and 2.

**Figure 3:**
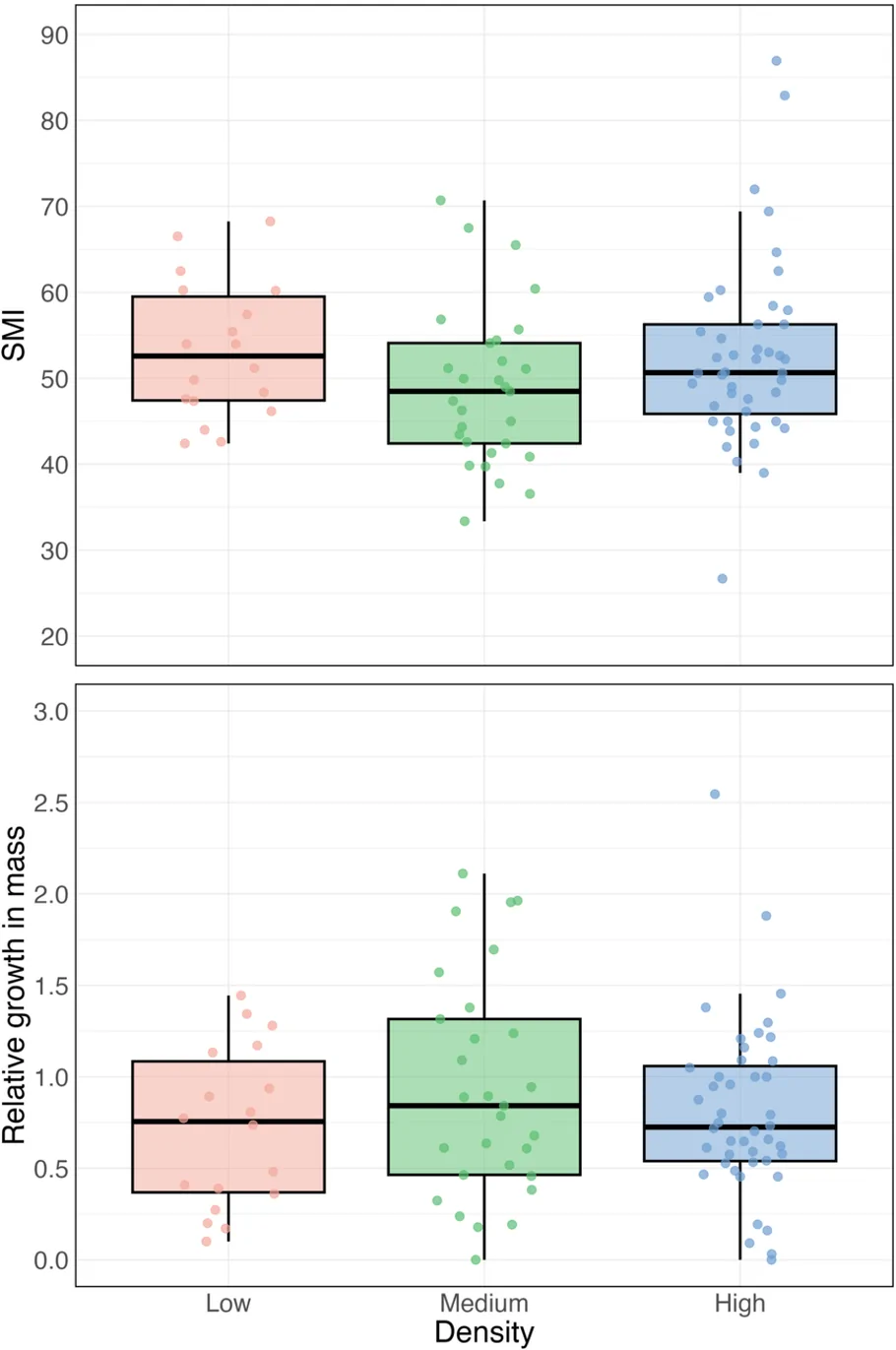
Box plots of relative growth in mass and scaled mass index (SMI) for Eastern Hellbenders (*Cryptobranchus alleganiensis alleganiensis*) reared at low (9 Hellbenders), medium (15 Hellbenders), or high (22 Hellbenders) density in Experiment 1. The data points represent each individual in the experiment.

### 3.2 Experiment 2. Interactive Effects of Density and Food Availability

We found significant main effects for food availability on relative growth in mass (*F*_2,120_ = 14.9, *p* < 0.001) but no effect of density (*F*_2,120_ = 1.5, *p* = 0.220) or the interaction (*F*_4,120_ = 1.0, *p* = 0.414). Based on Tukey HSD, relative growth in mass was 26%–46% greater in the high food availability treatment compared to both the low and medium food availability treatments (*p* ≤ 0.007; Figure 4). There was no difference between the low and medium food availability treatments (*p* = 0.184). For SMI, there was no effect of density (*F*_2,120_ = 1.1, *p* = 0.377), food availability (*F*_2,120_ = 0.2, *p* = 0.799), or their interaction (*F*_4,120_ = 0.3, *p* = 0.851). Across all treatments, SMI averaged 31.2 (Figure 4). The full dataset is available in the Supporting Information S1 and S2: Experiments 1 and 2.

**Figure 4:**
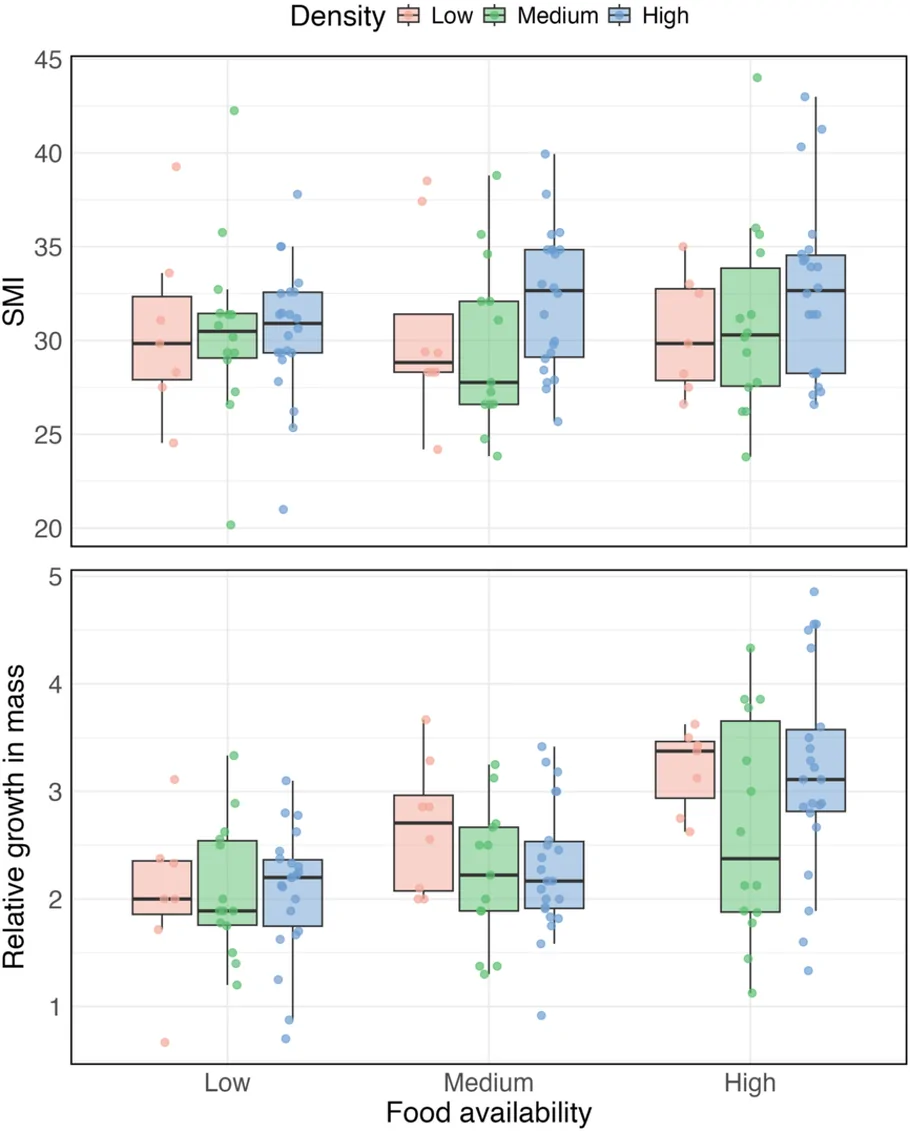
Box plots of relative growth in mass and scaled mass index (SMI) for Eastern Hellbenders (*Cryptobranchus alleganiensis alleganiensis*) in Experiment 2. The experiment was a factorial combination of density (low, medium, or high) and food availability (low, medium, or high). We added 4, 7, or 11 larvae per tank for low, medium, and high‐density treatments, respectively. The low, medium, and high food availability treatments were assigned a ration of 5.6%, 8.3%, and 11.1% of body mass per individual per feeding, respectively. The data points represent each individual in the experiment.

## 4 Discussion

This is the first study to evaluate and provide empirical data on the effects of density and food availability on growth rates and body condition for captive‐reared Hellbenders. Our findings demonstrate that Hellbenders can be reared at higher densities than current recommendations and that food availability can be a limiting factor. Importantly, these results suggest that husbandry practices can be modified to enhance conservation efforts for Hellbenders.

We found that food availability rather than density was the main driver of growth in our experiments. Surprisingly, we found that rearing Hellbenders at the highest densities yielded similar relative growth rates and SMI compared to individuals in lower densities. Moreover, the homogeneity tests for each experiment demonstrated that density did not influence the amount of variation in growth across the treatments. However, our study contrasts with Davis (2012), who suggested that large quantities of ambystomatid salamanders in captivity may limit the number of individuals available for release. Thus, the effects of density might need to be investigated at the species level prior to adopting the best approach.

Our data suggest that Hellbenders can be reared at higher densities than previously used without sacrificing growth rates. Increasing densities could have important implications for amphibian captive‐rearing programs (Iguchi et al. 2003; Procarione et al. 1999). For species that require stagnant systems (e.g., some tadpoles) or facilities that use recirculating systems, higher densities would produce more waste and could reduce water quality. However, our flow‐through system eliminates those concerns, as water is refreshed regularly. This is an important initial consideration for facilities when designing captive‐rearing systems for Hellbenders.

Rearing individuals at higher densities can lead to increased intraspecific aggression (Persson 1985), cannibalism (Slembrouck et al. 2009), and variation in body sizes (Nyman et al. 1993). We know that intraspecific aggression and cannibalism occur in Hellbenders (Unger and Williams 2018). In the current study, we documented a single injury resulting from intraspecific competition, and it was within the medium‐density treatment of the first experiment. We documented no cannibalism or attempted cannibalism. This is likely a result of both conservative densities and size‐sorting at the initiation of the study. Additionally, the mortalities that occurred during our study were scattered across treatments, suggesting that density was not the driver. Moreover, similarities in body condition among the treatments suggest that stress was not a concern because a general response to stress is a reduction in body condition. We recommend continual monitoring and size‐sorting for Hellbenders reared in captivity to prevent intraspecific aggression and cannibalism.

Another concern with higher densities is the potential consequences for disease management as most causes of disease in captivity are perceived to be either directly or indirectly linked to husbandry practices (Densmore and Green 2007; Poll 2009). Based on the necropsy results, the deaths during our studies were not associated with pathogens (e.g., ranavirus, *Batrachochytrium dendrobatidis*). While captive facilities should always be cognizant of disease threats, standard procedures for disinfection and decontamination should help to minimize potential outbreaks. Collectively, our data suggests that even the highest rearing densities in the current study may still be below optimal levels.

The second experiment showed that food levels have a significant impact on the relative growth of Hellbenders, suggesting that food is a limiting factor. While the low and medium food availability treatments were not significantly different, growth was significantly greater in the high treatment. This suggests that Hellbenders provided with food quantities representing at least 11.1% of their body mass will experience significant growth rates above current captive rearing guidelines. Given that food‐ restricted individuals often take longer to mature and mature at smaller body sizes (Dmitriew 2011; Manenti et al. 2023; Petranka and Sih 1986), increased food levels have the potential to substantially improve the output of Hellbender captive‐rearing programs. However, increased access to food and accelerated growth rates are not without potential drawbacks. Negative responses to high‐protein and/or fat diets and accelerated growth rates have been reported in captive fish, amphibians, and reptiles and include muscle hypertrophy and oxidative stress (Leandro et al. 2024), decreased survival (Harmon et al. 2023; Olsson and Shine 2002; Saikkonen et al. 2011), and a whole suite of potential physiological impairments (Park et al. 2025; Roh et al. 2020; Tamilarasan et al. 2025; Vargas and Vásquez 2017). Hellbenders are a long‐lived, relatively slowly maturing species, which could obscure our ability to observe the negative impacts associated with accelerated growth rates. It is important that the potential pros and cons of accelerated growth are considered while developing captive‐rearing plans. More research into the short and long‐term impacts of accelerated growth on captive‐reared Hellbenders is needed to further inform husbandry programs.

Thousands of Hellbenders are placed in captive‐rearing programs each year for conservation efforts, yet few published studies have examined rearing practices, and those that have are focused on the Ozark subspecies (Ettling et al. 2013). Inconsistent husbandry practices among rearing facilities could impact survival both during rearing and post‐release (Bodinof et al. 2012; Burgmeier et al. 2022; Kraus et al. 2017). Optimizing densities and food availability is crucial to maximizing conservation efforts while minimizing time spent in captivity. Current rearing guidelines typically produce release‐sized Hellbenders (minimum 50 g; (Briggler et al. 2014)) within a 3‐year rearing period. However, based on findings from the current study, if we maintain at least 11.1% food availability/body mass, we can increase densities by up to 47% with no negative impacts and can grow more Hellbenders above 50 g in a much shorter period. This increase could have profound impacts on time to restoration success and result in significant cost savings for project sponsors.

By increasing the density within Indiana's captive‐rearing programs alone, our projections indicate that we could complete planned releases up to 4 years sooner, while saving nearly $1,000,000 compared to our current management strategy. This would allow for more Hellbenders on the landscape, increasing the chances of establishing a stable population in Indiana and setting a precedent nationwide. We recommend that both nascent and existing Hellbender captive‐rearing programs consider increasing densities while developing plans for future release efforts. Implementing our results, in addition to improving other captive‐rearing standards (i.e., housing conditions), may result in larger translocated Hellbenders with a greater survival likelihood in nature.

## 5 Biosketch

The Help the Hellbender research team was formed in 2007 to advance the conservation of the Eastern Hellbender in Indiana. The team's efforts include a four‐phase initiative that integrates (1) research on Hellbender biology, spatial ecology, and health; (2) outreach and education; (3) captive rearing and release of Hellbenders; and (4) watershed‐scale habitat improvement. The team has partnered with a network of 27 organizations that includes federal and state agencies, local conservation districts, cattlemen's associations and producers, nonprofits, zoos, universities, and outreach organizations. More information can be found at helpthehellbender.org.

## Author Contributions

**Shelby M. Royal:** conceptualization, data curation, formal analysis, investigation, methodology, visualization, writing – original draft. **Summer S. Brown:** conceptualization, investigation, methodology, writing – original draft. **Nicholas G. Burgmeier:** conceptualization, funding acquisition, methodology, writing – review and editing. **Rod N. Williams:** conceptualization, data curation, funding acquisition, investigation, methodology, project administration, resources, supervision, writing – review and editing. **Jason T. Hoverman:** conceptualization, data curation, formal analysis, funding acquisition, methodology, visualization, writing – review and editing.

## Ethics Statement

All work was in accordance with protocols approved by the Purdue Animal Care and Use Committee (1406001094) and the IDNR (Indiana Scientific Purposes License 18‐017).

## Conflicts of Interest

The authors declare no conflicts of interest.

## Supporting information

Supporting File 1

Supporting File 2

## Data Availability

The data that support the findings of this study are available in the supporting material of this article.

## References

[zoo70087-bib-0001] Arbuckle, K. 2013. “Folklore Husbandry and a Philosophical Model for the Design of Captive Management Regimes.” Herpetological Review 44, no. 3: 448–452.

[zoo70087-bib-0002] Bodinof, C. M. , J. T. Briggler , R. E. Junge , et al. 2012. “Survival and Body Condition of Captive‐Reared Juvenile Ozark Hellbenders (*Cryptobranchus alleganiensis bishopi*) Following Translocation to the Wild.” Copeia 2012, no. 1: 150–159.

[zoo70087-bib-0003] Briggler, J. T. , T. L. Crabill , J. A. Civiello , et al. (2014). Population, Augmentation, and Reintroduction Plan for the Eastern Hellbender (*Cryptobranchus alleganiensis alleganiensis*) in Missouri. Eastern Hellbender Propagation Committee, Jefferson City, Missouri.

[zoo70087-bib-0004] Browne, R. K. , M. Pomering , and A. J. Hamer . 2003. “High Density Effects on the Growth, Development and Survival of *Litoria aurea* Tadpoles.” Aquaculture 215, no. 1–4: 109–121.

[zoo70087-bib-0005] Burgmeier, N. G. , J. D. Groves , N. Shields , et al. 2017. “How Our Zoos Help Hellbenders.” Purdue Extension 544: 1–16.

[zoo70087-bib-0006] Burgmeier, N. G. , E. B. McCallen , E. K. Kenison , and R. N. Williams . 2022. “Comparing the Effects of Environmental Enrichment, Seasonality, and Soft Release on Site Retention and Survivorship of Captive‐Reared Eastern Hellbenders (*Cryptobranchus alleganiensis alleganiensis*).” Herpetologica 78, no. 4: 225–234.

[zoo70087-bib-0007] Burgmeier, N. G. , S. D. Unger , J. L. Meyer , T. M. Sutton , and R. N. Williams . 2011. “Health and Habitat Quality Assessment for the Eastern Hellbender (*Cryptobranchus alleganiensis alleganiensis*) in Indiana USA.” Journal of Wildlife Diseases 47, no. 4: 836–848. 10.7589/0090-3558-47.4.836 22102654

[zoo70087-bib-0008] Van Buskirk, J. 2001. “Specific Induced Responses to Different Predator Species in Anuran Larvae.” Journal of Evolutionary Biology 14, no. 3: 482–489.

[zoo70087-bib-0009] Collins, J. P. , and J. E. Cheek . 1983. “Effect of Food and Density on Development of Typical and Cannibalistic Salamander Larvae in *Ambystoma tigrinum nebulosum* .” American Zoologist 23, no. 1: 77–84.

[zoo70087-bib-0010] Davis, A. K. 2012. “Investigating the Optimal Rearing Strategy for A*mbystoma* Salamanders Using a Hematological Stress Index.” Herpetological Conservation and Biology 7, no. 1: 95–100.

[zoo70087-bib-0011] Densmore, C. L. , and D. E. Green . 2007. “Diseases of Amphibians.” ILAR Journal 48, no. 3: 235–254. 10.1093/ilar.48.3.235 17592186

[zoo70087-bib-0012] Dmitriew, C. M. 2011. “The Evolution of Growth Trajectories: What Limits Growth Rate?” Biological Reviews 86, no. 1: 97–116. 10.1111/j.1469-185X.2010.00136.x 20394607

[zoo70087-bib-0013] Ettling, J. A. , M. D. Wanner , C. D. Schuette , S. L. Armstrong , A. S. Pedigo , and J. T. Briggler . 2013. “Captive Reintroduction and Husbandry of Adult Ozark Hellbenders, *Cryptobranchus alleganiensis bishopi* .” Herpetoculture 44: 605–610.

[zoo70087-bib-0014] Goldberg, T. , E. Nevo , and G. Degani . 2012. “Phenotypic Plasticity in Larval Development of Six Amphibian Species in Stressful Natural Environments.” Zoological Studies 51, no. 3: 345–361.

[zoo70087-bib-0015] Harmon, E. A. , T. Li , P. W. Kelly , C. Chen , D. W. Pfennig , and K. S. Pfennig . 2023. “A Maladaptive Parental Effect: Offspring Survival Decreases With Maternal Over‐Condition in an Amphibian.” Biological Journal of the Linnean Society 140, no. 4: 578–588.

[zoo70087-bib-0016] Harvey Pough, F. 2007. “Amphibian Biology and Husbandry.” ILAR Journal 48, no. 3: 203–213. 10.1093/ilar.48.3.203 17592184

[zoo70087-bib-0017] Iguchi, K. , K. Ogawa , M. Nagae , and F. Ito . 2003. “The Influence of Rearing Density on Stress Response and Disease Susceptibility of Ayu (*Plecoglossus altivelis*).” Aquaculture 220, no. 1–4: 515–523.

[zoo70087-bib-0018] Kaunert, M. D. , R. K. Brown , S. Spear , P. B. Johantgen , and V. D. Popescu . 2024. “Restoring Eastern Hellbender (Cryptobranchus a. alleganiensis) Populations Through Translocation of Headstarted Individuals.” Population Ecology 66, no. 2: 93–107.

[zoo70087-bib-0019] Kenison, E. K. , and R. N. Williams . 2018. “Rearing Captive Eastern Hellbenders (*Cryptobranchus a. alleganiensis*) With Moving Water Improves Swim Performance.” Applied Animal Behaviour Science 202: 112–118.

[zoo70087-bib-0020] Kraus, B. T. , E. B. McCallen , and R. N. Williams . 2017. “Evaluating the Survival of Translocated Adult and Captive‐Reared, Juvenile Eastern Hellbenders (*Cryptobranchus alleganiensis alleganiensis*).” Herpetologica 73, no. 4: 271–276.

[zoo70087-bib-0021] Leandro, S. V. , D. R. Ituassu , V. D. G. Sinhorin , et al. 2024. “Effect of Dietary Digestible Protein Levels on Muscle Growth and Oxidative Stress in Amazonian Pintado (*Pseudoplatystoma reticulatum* X *Leiarius marmoratus*).” Biology (Basel) 13, no. 10: 825. 10.3390/biology13100825 PMC11504720 39452134

[zoo70087-bib-0022] Luedtke, J. A. , J. Chanson , K. Neam , et al. 2023. “Ongoing Declines for the World's Amphibians in the Face of Emerging Threats.” Nature 622, no. 7982: 308–314. 10.1038/s41586-023-06578-4 PMC10567568 37794184

[zoo70087-bib-0023] Macke, M. J. , S. M. Royal , N. G. Burgmeier , B. J. Tornabene , and R. N. Williams . 2022. “Juvenile Eastern Hellbender (*Cryptobranchus a. allegaiviensis*) Preference Among Juvenile Hut Designs.” Herpetological Conservation and Biology 17, no. 2: 218–228.

[zoo70087-bib-0024] Macklem, D. C. , L. Augustine , M. D. Wanner , et al. 2025. “Growth and Mortality of Zoo‐Reared Ozark Hellbenders,Cryptobranchus alleganiensis bishopi(Grobman 1943).” Zoo Biology 44, no. 1: 72–86. 10.1002/zoo.21870 PMC11802483 39588554

[zoo70087-bib-0025] Manenti, R. , N. Kristensen , P. Cogliati , B. Barzaghi , A. Melotto , and G. F. Ficetola . 2023. “Larval Development and Poor Trophic Resource Availability: Local Adaptations and Plasticity in a Widespread Amphibian Species.” Journal of Evolutionary Biology 36, no. 3: 529–541. 10.1111/jeb.14155 36759955

[zoo70087-bib-0026] Morey, S. , and D. Reznick . 2001. “Effects of Larval Density on Postmetamorphic Spadefoot Toads (*Spea hammondii*).” Ecology 82, no. 2: 510–522.

[zoo70087-bib-0027] Newman, R. A. 1994a. “Effects of Changing Density and Food Level on Metamorphosis of a Desert Amphibian, *Scaphiopus couchii* .” Ecology 75, no. 4: 1085–1096.

[zoo70087-bib-0028] Newman, R. A. 1994b. “Genetic‐Variation for Phenotypic Plasticity in the Larval Life‐History of Spadefoot Toads (*Scaphiopus couchii*).” Evolution 48, no. 6: 1773–1785. 10.1111/j.1558-5646.1994.tb02213.x 28565166

[zoo70087-bib-0029] Nyman, S. , R. F. Wilkinson , and J. E. Hutcherson . 1993. “Cannibalism and Size Relations in a Cohort of Larval Ringed Salamanders (*Ambystoma cnnulatum*).” Journal of Herpetology 27, no. 1: 78–84.

[zoo70087-bib-0030] Olsson, M. , and R. Shine . 2002. “Growth to Death in Lizards.” Evolution 56, no. 9: 1867–1870. 10.1111/j.0014-3820.2002.tb00202.x 12389733

[zoo70087-bib-0031] Park, J. , Y. Lee , J. Y. Lee , et al. 2025. “Overfeeding in Rainbow Trout (*Oncorhynchus mykiss*): Metabolic Disruptions, Impaired Immunity, and Increased Infection Risk.” Fish & Shellfish Immunology 160: 110224. 10.1016/j.fsi.2025.110224 39988219

[zoo70087-bib-0032] Peig, J. , and A. J. Green . 2009. “New Perspectives for Estimating Body Condition From Mass/Length Data: The Scaled Mass Index as an Alternative Method.” Oikos 118, no. 12: 1883–1891.

[zoo70087-bib-0033] Persson, L. 1985. “Asymmetrical Competition ‐ Are Larger Animals Competitively Superior.” American Naturalist 126, no. 2: 261–266.

[zoo70087-bib-0034] Petranka, J. W. 1989. “Density‐Dependent Growth and Survival of Larval *Ambystoma* ‐ Evidence From Whole‐Pond Manipulations.” Ecology 70, no. 6: 1752–1767.

[zoo70087-bib-0035] Petranka, J. W. , and A. Sih . 1986. “Environmental Instability, Competition, and Density‐Dependent Growth and Survivorship of a Stream‐Dwelling Salamander.” Ecology 67, no. 3: 729–736.

[zoo70087-bib-0036] Poll, C. P. 2009. “Wound Management in Amphibians: Etiology and Treatment of Cutaneous Lesions.” Journal of Exotic Pet Medicine 18, no. 1: 20–35.

[zoo70087-bib-0037] Procarione, L. S. , T. P. Barry , and J. A. Malison . 1999. “Effects of High Rearing Densities and Loading Rates on the Growth and Stress Responses of Juvenile Rainbow Trout.” North American Journal of Aquaculture 61, no. 2: 91–96.

[zoo70087-bib-0038] R Development Core Team . 2024. R: A Language and Environment for Statistical Computing. R Foundation for Statistical Computing. http://www.R-project.org.

[zoo70087-bib-0039] Relyea, R. A. 2001. “Morphological and Behavioral Plasticity of Larval Anurans in Response to Different Predators.” Ecology 82, no. 2: 523–540.

[zoo70087-bib-0040] Relyea, R. A. 2002. “Competitor‐Induced Plasticity in Tadpoles: Consequences, Cues, and Connections to Predator‐Induced Plasticity.” Ecological Monographs 72, no. 4: 523–540.

[zoo70087-bib-0041] Relyea, R. A. , and J. R. Auld . 2004. “Having the Guts to Compete: How Intestinal Plasticity Explains Costs of Inducible Defences.” Ecology Letters 7, no. 9: 869–875.

[zoo70087-bib-0042] Relyea, R. A. , and J. R. Auld . 2005. “Predator‐ and Competitor‐Induced Plasticity: How Changes in Foraging Morphology Affect Phenotypic Trade‐Offs.” Ecology 86, no. 7: 1723–1729.

[zoo70087-bib-0043] Roh, H. , J. Park , A. Kim , et al. 2020. “Overfeeding‐Induced Obesity Could Cause Potential Immuno‐Physiological Disorders in Rainbow Trout (*Oncorhynchus mykiss*).” Animals: An Open Access Journal from MDPI 10, no. 9: 1499. 10.3390/ani10091499 PMC7552159 32854279

[zoo70087-bib-0044] Saikkonen, A. , J. Kekäläinen , and J. Piironen . 2011. “Rapid Growth of Atlantic Salmon Juveniles in Captivity May Indicate Poor Performance in Nature.” Biological Conservation 144, no. 9: 2320–2327.

[zoo70087-bib-0045] Sannolo, M. , F. Gatti , M. Mangiacotti , S. Scali , and R. Sacchi . 2016. “Photo‐Identification in Amphibian Studies: A Test of I^3^S Pattern.” Acta Herpetologica 11, no. 1: 63–68.

[zoo70087-bib-0046] Scott, J. M. , D. D. Goble , J. A. Wiens , D. S. Wilcove , M. Bean , and T. Male . 2005. “Recovery of Imperiled Species Under the Endangered Species Act: The Need for a New Approach.” Frontiers in Ecology and the Environment 3, no. 7: 383–389.

[zoo70087-bib-0047] Slembrouck, J. , E. Baras , J. Subagja , L. T. Hung , and M. Legendre . 2009. “Survival, Growth and Food Conversion of Cultured Larvae of *Pangasianodon hypophthalmus*, Depending on Feeding Level, Prey Density and Fish Density.” Aquaculture 294, no. 1–2: 52–59.

[zoo70087-bib-0048] Stuart, S. N. , J. S. Chanson , N. A. Cox , et al. 2004. “Status and Trends of Amphibian Declines and Extinctions Worldwide.” Science 306, no. 5702: 1783–1786. 10.1126/science.1103538 15486254

[zoo70087-bib-0049] Tamilarasan, S. , B. J. Pratima , and P. R . 2025. “Understanding the Intense Effects of Caloric Restriction and Overfeeding on Zebrafish (*Danio rerio*) Behaviour and Biochemical Processes, Along With Their Implications for Offspring.” Journal of Clinical Medicine & Health Care 2: 1–10.

[zoo70087-bib-0050] Tornabene, B. J. , and J. T. Hoverman . 2018. “The Salamander Daisy: A Novel Captive Rearing Method for Cannibalistic Salamander Larvae.” Herpetological Conservation and Biology 13, no. 1: 167–174.

[zoo70087-bib-0051] Unger, S. D. , and R. N. Williams . 2018. “Genetic Confirmation of Filial Cannibalism in North America's Giant Salamander, the Eastern Hellbender *Cryptobranchus alleganiensis alleganiensis* .” Ethology Ecology & Evolution 30, no. 2: 187–193.

[zoo70087-bib-0052] Vargas, R. , and I. C. Vásquez . 2017. “Effects of Overfeeding and High‐Fat Diet on Cardiosomatic Parameters and Cardiac Structures in Young and Adult Zebrafish.” Fish Physiology and Biochemistry 43, no. 6: 1761–1773. 10.1007/s10695-017-0407-7 28842785

[zoo70087-bib-0053] Wildy, E. L. , D. P. Chivers , J. M. Kiesecker , and A. R. Blaustein . 2001. “The Effects of Food Level and Conspecific Density on Biting and Cannibalism in Larval Long‐Toed Salamanders, *Ambystoma macrodactylum* .” Oecologia 128, no. 2: 202–209. 10.1007/s004420100641 28547469

[zoo70087-bib-0054] Williams, R. D. , J. E. Gates , C. H. Hocutt , and G. J. Taylor . 1981. “The Hellbender *Cryptobranchus alleganiensis alleganiensis* a Nongame Species in Need of Management.” Wildlife Society Bulletin 9, no. 2: 94–100.

